# Talin-1 interaction network promotes hepatocellular carcinoma progression

**DOI:** 10.18632/oncotarget.14674

**Published:** 2017-01-16

**Authors:** Peng Chen, Xiaohu Zheng, Yonggang Zhou, Yechuan Xu, Lixin Zhu, Yeben Qian

**Affiliations:** ^1^ Department of General Surgery, First Affiliated Hospital of Anhui Medical University, Hefei, China; ^2^ Institute of Immunology and the CAS Key Laboratory of Innate Immunity and Chronic Disease, School of Life Sciences and Medical Center, University of Science and Technology of China, Hefei, China; ^3^ Center Laboratory, First Affiliated Hospital of Anhui Medical University, Hefei, China

**Keywords:** Talin-1, hepatocellular carcinoma, tumor growth and metastasis, ion transport, membrane depolarization

## Abstract

Talin-1 is a known oncogene-associated protein. In this study, we set out to determine its role and mechanisms in hepatocellular carcinoma (HCC) progression. We found Talin-1 to be highly expressed in HCC cells relative to non-cancer liver epithelial cells and to promote tumor growth and metastasis. We used Whole Human Genome Oligo Microarray analysis with HCC cells and HCC cells in which Talin-1 was knocked down using shRNA to identify transcripts regulated by Talin-1. Of the 40,000 tested genes, 3099 were differentially expressed after Talin-1 knockdown; expression of 1924 genes was increased, while expression of 2175 was decreased. Gene ontology (GO) profiling indicated that Talin-1 promotes many HCC progression-related activities, including ion transport and membrane depolarization, cell growth, and cell adhesion. We also characterized the network of gene transcripts regulated by Talin-1 and their role in promoting HCC progression. Our findings confirm the role of Talin-1 in carcinogenesis and provided a set of novel therapeutic targets for the treatment of HCC.

## INTRODUCTION

Hepatocellular carcinoma (HCC) is a common malignancy of the digestive system [1]. Patients are often diagnosed with HCC at an advanced stage, and show a poor prognosis [2]. Therefore, novel diagnostic and therapeutic methods are needed for early cancer detection and effective treatment for this large group of patients.

Talin-1, a macromolecular cytoskeletal protein, has been reported to interact with multiple adhesion molecules (e.g. integrin and F-actin) and to activate the integrin/focal adhesion kinase (FAK) pathway [3, 4]. Studies indicated that Talin-1 is a potential marker for diagnosing cancer at an early stage because its high expression level in serum specimens from cancer patients was sufficient to distinguish them from normal human samples [5, 6]. Specifically, Youns MM et al. found that sensitivity and specificity of Talin-1 for diagnosing cancer were higher than those of alpha-fetoprotein (AFP) in Egyptian HCC patients [5, 6]. Overall, these findings suggested that Talin-1 is a potential diagnostic marker for HCC. However, whether Talin-1 promoted HCC growth and metastasis was still uncertain, and the mechanisms of Talin-1 in HCC progression remained unclear.

In this study, we assessed the proliferation and migration abilities of Talin-1 knockdown and normal control HCC cells. We used human whole-genome microarray assays and GO profiling to examine the relation between Talin-1-regulated gene transcripts and HCC progression-related biological behaviors. Furthermore, combining microarray data and online bioinformatics, we sought to define the interaction network (downstream genes and pathways) of Talin-1 in HCC.

## RESULTS

### Talin-1 promotes HCC growth and metastasis

**Figure 1:**
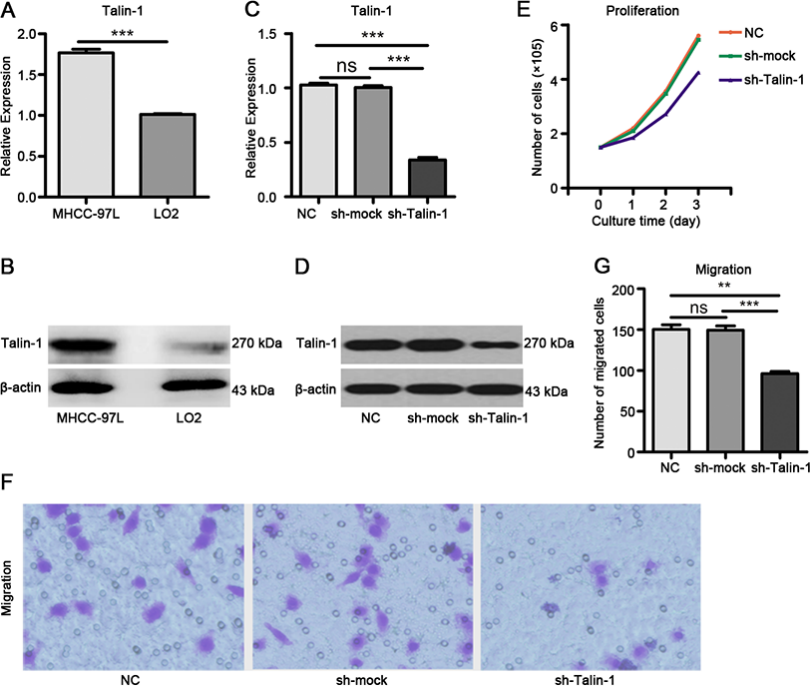
Talin-1 promotes HCC growth and metastasis Talin-1 is more highly expressed in MHCC-97L cells compared with LO2 cells in terms of (**A**) mRNA levels and (**B**) protein amounts. Talin-1 expression is lower in the sh-Talin-1 group than the NC and sh-mock groups in (**C**) mRNA levels and (**D**) protein amounts. (**E**) Cell proliferation was assessed by counting. Markedly fewer cells were found in the sh-Talin-1 group compared with NC and sh-mock groups at 24 h, 48 h or 72 h; (**F, G**), HCC cell migration ability was assessed by transwell migration assay. Migration was significantly reduced in the sh-Talin-1 group compared with the NC and sh-mock groups. *P < 0.05 **P < 0.01, and ***P < 0.001. The data are presented as the mean SEM.

We selected a HCC cell line (MHCC-97L cells) and a normal liver epithelial cell line (LO2 cells) to analyze the function of Talin-1 in HCC. We found Talin-1 mRNA (Figure 1A) (*P <* 0.0001) and protein (Figure 1B) levels were higher in MHCC-97L cells compared with LO2 cells. Next, we established a stable Talin-1 knockdown MHCC-97 L cell line. qRT-PCR (Figure 1C) and Western blot (Figure 1D) at 72 h after transduction confirmed that both Talin-1-shRNA and scramble shRNA were successfully and efficiently transfected into cells. Talin-1 mRNA levels in cells transduced with Talin-1-shRNA (sh-Talin-1 group) were overtly decreased compared with the amounts obtained after transduction with scramble shRNA (sh-mock group) and non-transduced cells (negative control group, NC group) (both *P <* 0.0001). Cell proliferation and metastasis assays were performed on sh-Talin-1, NC, and sh-mock groups. By cell counting, we found markedly fewer cells in the sh-Talin-1 group compared with NC and sh-mock groups at 24 h (*p* = 0.0147 and 0.0114, respectively), 48 h (*p* = 0.0006 and < 0.0001, respectively) and 72 h (both *P <* 0.0001) (Figure 1E). In transwell assay, migration was significantly reduced in the sh-Talin-1 group compared with the NC and sh-mock groups (*p* = 0.001 and 0.0007, respectively) (Figure 1F, 1G). Taken together, these findings suggest that Talin-1 promotes HCC growth and metastasis.

### The biological behaviors regulated by Talin-1 in promoting HCC

**Figure 2:**
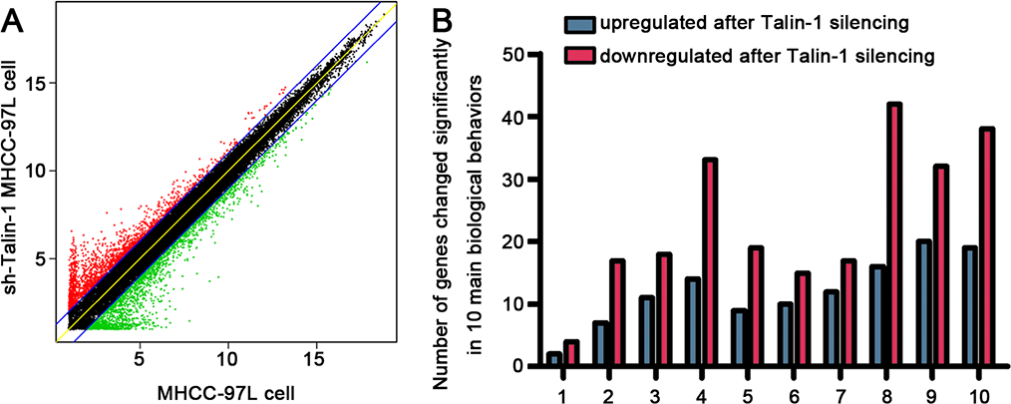
The biological behaviors regulated by Talin-1 in promoting HCC (**A**), Scatter plot of genes changed by twofold or more after Talin-1 silencing in HCC. Dots highlighted in red and green represent the 2-fold up-regulated and down-regulated genes after Talin-1 silencing, respectively. (**B**) 10 main biological behaviors affected by Talin-1 in HCC.

To explore the downstream targets of Talin-1 in HCC progression, we performed high-resolution microarray analysis (a total of 40000 genes) on the NC and sh-Talin-1 groups (GEO serial number: GSE86062). As shown in Figure 2A, of the 40000 tested genes, 3099 were differentially expressed after Talin-1 knockdown (1924 genes increased and 2175 genes dropped). To further assess the biological behaviors regulated by Talin-1in HCC, the 3099 genes were classified into categories according to GO annotation (Figure 2B). The top 10 dramatically changed biological behaviors ranked by enrichment factor are listed in Table 1. They included membrane repolarization-related (enrichment factor = 7.611999094), cell growth-related (enrichment factor = 2.282109371), ion transport-related (enrichment factor = 1.792892772), extracellular space-related (enrichment factor = 1.750628487), cell adhesion-related (enrichment factor = 1.729052887), transporter activity-related (enrichment factor = 1.693649687), transmembrane transport-related (enrichment factor = 1.689015481), regulation of multicellular organism processes-related (enrichment factor = 1.564495121), plasma membrane-related (enrichment factor = 1.551785285), and response to external stimulus-related (enrichment factor = 1.52078304)genes; the 4 most prominent categories were selected for detailed analysis, i.e. ion transport, membrane depolarization, cell growth, and cell adhesion.

**Table 1:** GO profiling identified in 10 main biological behaviors after Talin-1 silencing

GO_term (ID)	*P*-value	enrich_factor	Nr. Genes (upregulate/downregulate)	up-regulated geneID (mRNA)	down-regulated geneID (mRNA)
membrane repolarization	1.74E-06	7.611999094	6 (2/4)	KCNE3 ([NM_005472]),	GJA1 ([NM_000165]), KCNQ1 ([NM_000218]),
(GO:0086009)				CACNA1D ([NM_000720])	AKAP6 ([NM_004274]), CAV3 ([NM_001234])
cell growth	5.37E-06	2.282109371	24 (7/17)	CGREF1 ([NM_006569]), SLC44A4 ([NM_025257]),	EDN1 ([NM_001955]), IGFBP5 ([NM_000599]),
(GO:0016049)				RIMS1 ([NM_014989]), TRIM40 ([NM_138700]),	CYR61 ([NM_001554]), PDGFB ([NM_002608]),
				OMG ([NM_002544]), SLIT2 ([NM_004787])	CTGF ([NM_001901]), IGFBP3 ([NM_001013398])
ion transport	2.50E-08	1.792892772	29 (11/18)	KCNE3 ([NM_005472]), SLCO1B3 ([NM_019844]),	CLIC3 ([NM_004669]), CTGF ([NM_001901]),
(GO:0006811)				ALB ([NM_000477]), TESC ([NM_017899])	CAV3 ([NM_001234]), AQP1 ([NM_198098]),
					PTGS2 ([NM_000963]), CA9 ([NM_001216]),
extracellular space	2.73E-07	1.750628487	47 (14/33)	MUC5B ([NM_002458]), MMEL1 ([NM_033467]),	EDN1 ([NM_001955]), WISP2 ([NM_003881]),
(GO:0005615)				SLIT2 ([NM_004787]), SPX ([NM_030572]),	KRT34 ([NM_021013]), MUC17 ([NM_001040105]),
				PDGFC ([NM_016205]), FAM184A ([NM_024581])	KLHL34 ([NM_153270]), VTN ([NM_000638])
cell adhesion	6.06E-07	1.729052887	28 (9/19)	VCAM1 ([NM_001078]), ADAM22 ([NM_021721]),	MUC1 ([NM_002456]), SLAMF7 ([NM_021181]),
(GO:0007155)				RGCC ([NM_014059]), CGREF1 ([NM_006569]),	WISP1 ([NM_003882]), VTN ([NM_000638]),
					GLI2 ([NM_005270]), FGB ([NM_005141])
transporter activity	8.73E-06	1.693649687	25 (10/15)	SLCO1B3 ([NM_019844]), ANO5 ([NM_213599]),	PTGDS ([NM_000954]), CLIC3 ([NM_004669]),
(GO:0005215)				SLC26A9 ([NM_052934]), KCNE3 ([NM_005472]),	PDE2A ([NM_002599]), SLC2A14 ([NM_001286233]),
				SLC39A8 ([NM_022154]), SCN9A ([NM_002977])	ANKH ([NM_054027]), SLC2A5 ([NM_003039])
transmembrane transport	4.51E-06	1.689015481	29 (12/17)	SLCO1B3 ([NM_019844]), ANO5 ([NM_213599]),	CLIC3 ([NM_004669]), PDE2A ([NM_002599]),
(GO:0055085)				ALB ([NM_000477]), SLC26A9 ([NM_052934]),	CAV3 ([NM_001234]), EDN1 ([NM_001955]),
				TESC ([NM_017899]), KCNE3 ([NM_005472])	SLC2A14 ([NM_001286233]), ANKH ([NM_054027])
regulation of multicellular organismal process	1.26E-07	1.564495121	58 (16/42)	IFITM1 ([NM_003641]), SLIT2 ([NM_004787]),	CYR61 ([NM_001554]), EDN1 ([NM_001955]),
(GO:0051239)				SEMA4D ([NM_006378]), SPX ([NM_030572]),	NR4A2 ([NM_006186]), ANKH ([NM_054027]),
				TESC ([NM_017899]), ADM2 ([NM_024866])	EGR3 ([NM_004430]), GDNF ([NM_000514])
plasma membrane part	2.43E-08	1.551785285	52 (20/32)	FUT1 ([NM_000148]), SLCO1B3 ([NM_019844]),	CD247 ([NM_198053]), DTNA ([NM_001128175]),
(GO:0044459)				TSPAN32 ([NM_139022]), CEACAM5 ([NM_004363]),	PDE2A ([NM_002599]), CAV3 ([NM_001234]),
				SLC26A9 ([NM_052934]), TESC ([NM_017899])	ANKH ([NM_054027]), DUOX2 ([NM_014080])
response to external stimulus	4.78E-07	1.52078304	57 (19/38)	MATN2 ([NM_030583]), MUC5B ([NM_002458]),	FGFBP1 ([NM_005130]), DUOX2 ([NM_014080]),
(GO:0009605)				IFITM1 ([NM_003641]), SLIT2 ([NM_004787]),	NR4A1 ([NM_002135]), VTN ([NM_000638]),
				UNC5B ([NM_170744]), NPAS3 ([NM_022123])	TREM1 ([NM_018643]), FOSB ([NM_006732])

### Talin-1 promotes ion transport and membrane depolarization in HCC

**Figure 3:**
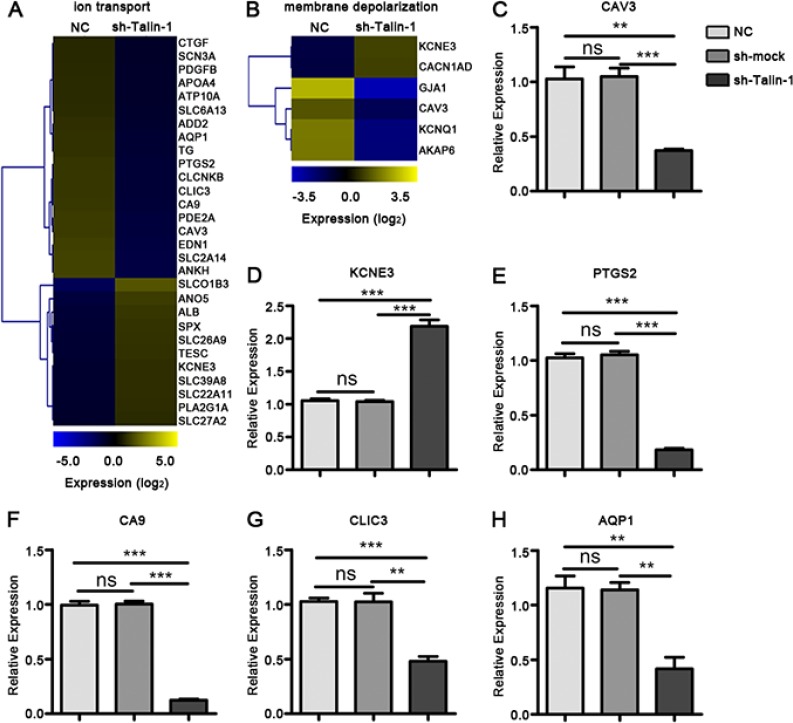
Talin-1 promotes ion transport and membrane depolarization in HCC (**A, B**) Heat maps of genes changed by two fold or more after Talin-1 knockdown involved in (A) ion transport and (B) membrane depolarization. The signal value of the genes is higher in yellow box than in blue ones. (**C–G**) Expression of selected genes involved in ion transport and membrane depolarization are measured by qRT-PCR. (C) CAV3, (H) AQP1, (E) PTGS2, (F) CA9 and (G) CLIC3 were down-regulated while (D) KCNE3 was up-regulated in the sh-Talin-1 group compared with the NC and sh-smock groups. *P < 0.05 **P < 0.01, and ***P < 0.001. The data are presented as the mean ± SEM.

Ion transport, a primary tumor cell migration regulator, can cause membrane depolarization-dependent promotion of cell proliferation [7–9]. Via GO assay, Talin-1 was found to promote both ion transport and membrane depolarization. Ion transport regulates membrane depolarization, and most genes present in one pathway are found in both; thus, the two categories were assessed together. Heat maps were generated using microarray data to further clarify changes in expression of genes involved in ion transport (Figure 3A) and membrane depolarization (Figure 3B) after Talin-1 silencing. The major ion transport- and depolarization-related genes were downregulated after Talin-1 silencing. The number of downregulated ion transport-related genes (18 genes) was markedly higher than that of upregulated ones (11 genes) (Figure 3A), and the change in membrane depolarization-related genes had the same tendency, with the number of downregulated genes twice the number of upregulated ones (Figure 3B). Overall, these indicate that Talin-1 may promote ion transport and membrane depolarization.

Ca^2+^ influx resulting in voltage-gated Ca^2+^ (Cav) channel-mediated current triggers membrane depolarization, while K^+^ efflux and Cl^-^ influx are the principle ion currents that attenuate membrane depolarization [7–9]. Caveolin 3 (CAV3) could significantly attenuate the opioid receptor-mediated inhibition of N-type Ca^2+^ channels and increase Ca^2+^ influx [10, 11]. Meanwhile, potassium voltage-gated channel subfamily E regulatory subunit 3 (KCNE3) was reported to increase K^+^ efflux and Cl^-^ influx [12, 13]. Combining the data of our microarray and verified PCR, we found that, after Talin-1 silencing CAV3 declined significantly (Figure 3A–3C), while KCNE3 was significantly upregulated (Figure 3A, 3B, 3D). Overall, Talin-1 may promote membrane depolarization by increasing Ca^2+^ influx and inhibiting K^+^ efflux and Cl^-^ influx. Additionally, other PCR verified ion transport- and depolarization-related genes, including prostaglandin-endoperoxide synthase 2 (PTGS2) (Figure 3A, 3E), carbonic anhydrase 9 (CA9) (Figure 3A, 3F), chloride intracellular channel 3 (CLIC3) (Figure 3A, 3G), and aquaporin 1 (AQP1) (Figure 3A, 3H), decreased in the sh-Talin-1 group compared with the NC groups (*P <* 0.0001, < 0.0001, = 0.0005 and = 0.0084, respectively) and sh-smock (*P <* 0.0001, < 0.0001, = 0.0038 and = 0.0044, respectively).

### Talin-1 promotes cell growth and upregulates cell cycle related genes in HCC

**Figure 4:**
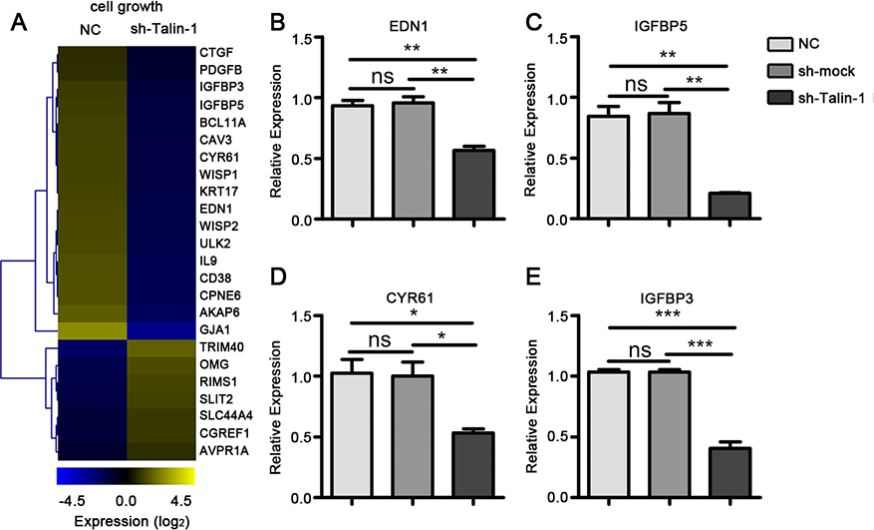
Talin-1 promotes cell growth in HCC (**A**) The heat map of cell growth-related genes changed by two fold or more after Talin-1 silencing in HCC. The signal value of the genes is higher in the yellow box than in blue ones; (**B–E**), Expression of selected cell growth-related genes measured by qRT-PCR. (B) EDN1, (C) IGFBP5, (D) CYR61 and (E) IGFBP3 were downregulated in the sh-Talin-1 group compared with the NC and sh-mock groups. *P < 0.05 **P < 0.01, and ***P < 0.001. The data are presented as the mean ± SEM.

Unchecked growth and apoptosis avoidance are hallmarks of cancer cells [14]. We found that a large number of cell growth genes changed after Talin-1 knockdown using data from the GO project. To clarify the changes of cell growth-related genes after Talin-1 silencing, a heat map was generated with the microarray data using the MEV 4.9 software. According to Figure 4A, the expression levels of 24 cell growth-related genes were overtly changed after Talin-1 silencing, and the number of raised genes (17 genes) was more than double that of reduced ones (7 genes). Overall, the data of the heat map indicates that Talin-1 may promote cell growth.

Endothelin-1 (EDN1) and insulin-like growth factor binding protein 5 (IGFBP5) are well-known cell cycle regulators in the p53 network, and they are reported to be highly expressed in HCC [15, 16]. Combining the data of our microarray and PCR, we found that the expression of both EDN1 (Figure 4A, 4B) and IGFBP5 (Figure 4A, 4C) was diminished after Talin-1 silencing. Meanwhile, cysteine-rich angiogenic inducer 61 (CYR61), a growth factor-inducible gene, is reported to promote cell proliferation by decreasing p21, p53, and Bax expression levels and increasing Bcl-xL, Mcl-1, Bcl-2 and NF-κB [17, 18]. We also found that the expression of CYR61 was downregulated after Talin-1 knockdown (Figure 4A, 4D). These findings suggest that Talin-1 promotes cell growth by regulating the p53 network and BCL-2 family. Additionally, the expression of insulin-like growth factor binding protein 3 (IGFBP3), a member of insulin-like growth factor family, was downregulated in the sh-Talin-1 group compared with the NC and sh-mock groups (Figure 4A, 4E).

### Talin-1 promotes cell adhesion in HCC

**Figure 5:**
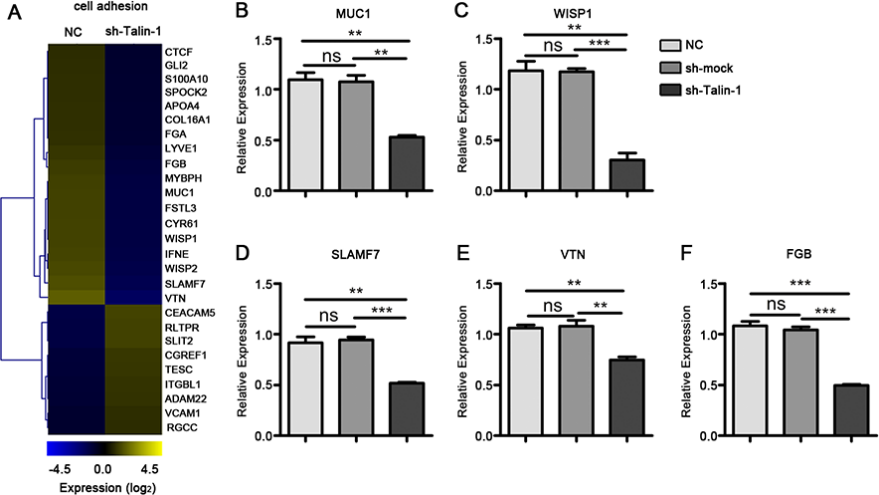
Talin-1 regulates cell adhesion in HCC (**A**) The heat map of genes changed by two fold or more in cell adhesion after Talin-1 silencing in HCC. The signal value of the genes is higher in yellow box than in blue ones; (**B–F**) Expression of selected genes in cell adhesion measured by qRT-PCR. (B) MUC1, (C) WISP1, (D) SLAMF7, (E) VTN and (F) FGB were down-regulated in the sh-Talin-1 group compared with the NC and sh-mock groups. *P < 0.05 **P < 0.01, and ***P < 0.001. The data are presented as the mean SEM.

Adhesion is essential to the metastatic spread of tumor cells. Using GO profiling, we found that Talin-1 regulated many cell adhesion-related genes. To assess changes of cell adhesion-related genes after Talin-1 silencing, we generated a heat map using the microarray data (Figure 5A). According to the heat map, the expression levels of 28 cell adhesion-related genes were altered by ≥ 2 fold, and the number of downregulated genes (19 genes) was more than double that of upregulated ones (9 genes), which indicates that Talin-1 may promote cell adhesion.

Epithelial-to-mesenchymal transition (EMT) refers to the shift by epithelial cells to a mesenchymal phenotype in morphology and acquisition of metastasis markers [19]. Mucin 1 (MUC1) and WNT1 inducible signaling pathway protein 1 (WISP1) have been reported to inhibit E-cadherin-mediated cell-cell adhesion, induce the expression of transcription factors (Snail, N-cadherin, ZEB1 and Smad2), and in turn activate EMT in cancer cells [20, 21]. Combining the data of our microarray and verified PCR, we found that both MUC1 (Figure 5A, 5B) and WISP1 (Figure 5A, 5C) decreased after Talin-1 knockdown. Overall, these data suggest that Talin-1 may promote cell adhesion via regulating the EMT process. Interestingly, WISP1 was also shown to promote tumor cell metastasis by activating PI3K/Akt/mTOR signaling [20, 21], which indicates that Talin-1 may promote cell adhesion via the classic mTOR pathway. Additionally, other PCR verified cell adhesion-related genes including SLAM family member 7 (SLAMF7) (Figure 5A, 5D), vitronectin (VTN) (Figure 5A, 5E), and fibrinogen beta chain (FGB) (Figure 5A, 5F) were also downregulated in the sh-Talin-1 group compared with the NC (*P* = 0.0022, 0.0018 and 0.0002, respectively) and sh-mock (*P* = 0.0002, 0.0070 and < 0.0001, respectively).

Taken together, these findings indicate that Talin-1 enhances cell adhesion. Furthermore, Talin-1 enhanced a wide range of biological behaviors, including ion transport, membrane depolarization, cell growth, and cell adhesion, which can drastically promote HCC genesis and progression.

### Interaction network of Talin-1

The genes regulated by Talin-1 may play important roles in HCC phenotypes, including growth, metastasis, and apoptosis. However, identifying their downstream targets or upstream regulators remains challenging.

**Figure 6:**
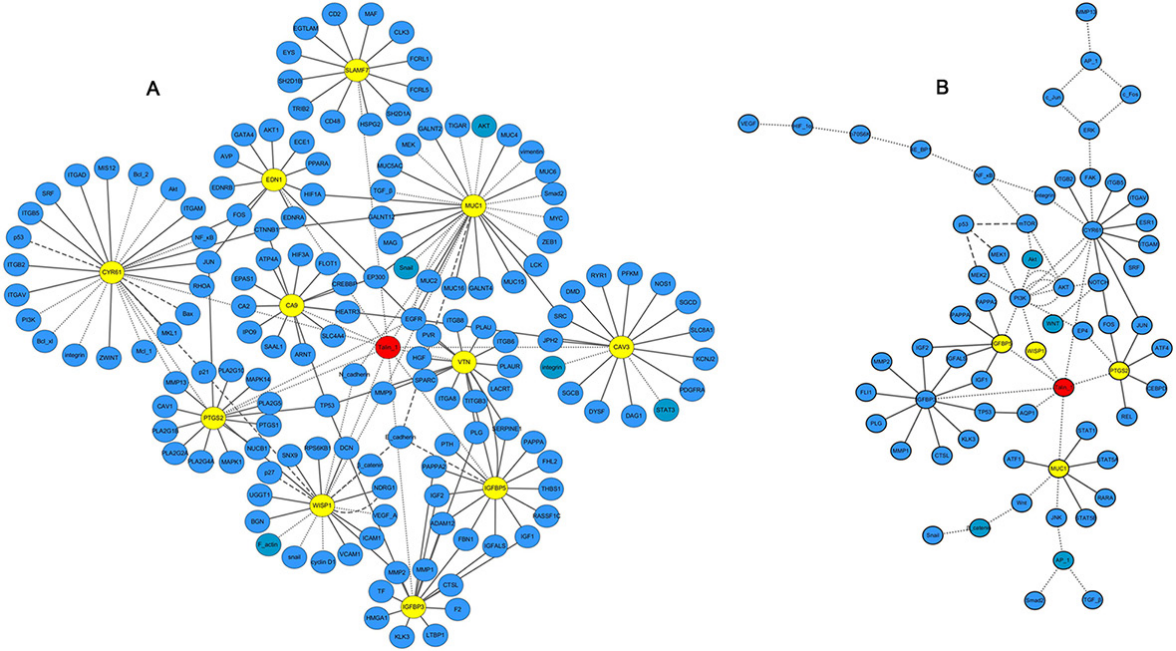
Interaction network of Talin-1 in HCC (**A**) putative physical and regulatory interactions between Talin-1 and candidate transcriptional regulators, and between the later and their target genes in HCC; (**B**) pathways regulated by Talin-1 and candidate transcriptional regulators in HCC. Talin-1 is in red; the 11 selected genes are in yellow; the other searched genes are in blue.

Based on the above microarray data validated by qRT-PCR, 11 prominent genes (SLAMF7, MUC1, CAV3, VTN, IGFBP3, IGFBP5, WISP1, PTGS2, CA9, EDN1, and CYR61) were selected from the four cell biological behaviors identified here, as potential candidate targets of Talin-1 in HCC. Next, we searched for and predicted putative target genes of all the 11 genes by combining information from two bioinformatics databases (STRING and GeneMANIA) and published studies. Finally, the bioinformatics software Cytoscape v3.4.0 was used to reconstruct the potential interaction network of Talin-1 in promoting HCC, including molecular and pathway networks (Figure 6). In the resulting molecular network (Figure 6A) Talin-1 is presented in red and the genes in yellow form the remaining 11 network genes, and their downstream targets. Additionally, cell growth and apoptosis are mainly regulated by the BCL-2 family and p53 network [22]. The BCL-2 family includes apoptotic (Bax, Bak, Bid and Bim) and anti-apoptotic (Bcl-2, Bcl-xl and Bcl-w) members [23]. Meanwhile, p53 and p21 are well-known apoptosis factors in the p53 network [24]. In the network proposed here (Figure 6A), we found that Talin-1 could inhibit the expression of TP53, p21, and Bax, and increase the expression of Bcl-xl and Bcl-2. Further, as shown in Figure 6A, Talin-1 enhances the expression of EMT inducer genes like Snail, N-cadherin, and vimentin, while suppressing the expression of E-cadherin. Overall, the data suggest that Talin-1 promotes growth and metastasis via regulating its potential targets (BCL-2 family, p53 network, EMT process). Finally, the resulting pathway network (Figure 6B) showed that Talin-1 could activate the PI3K/Akt/ mTOR pathway, NF-κB pathway, IGFR/IGF axis, and Wnt pathway. These findings suggest that Talin-1 regulates a complex network in HCC to promote cell growth and metastasis.

## DISCUSSION

In this study, we found that Talin-1 could markedly promote HCC proliferation and metastasis. Additionally, by using whole-genome microarray analysis and GO profiling, we found that Talin-1-regulated biological behaviors which could induce HCC progression, including ion transport, membrane repolarization, cell growth, and cell adhesion. Finally, in combination with microarray data, we searched published reports and online bioinformatics databases and generated an interaction network (genes and pathways) for Talin-1 in HCC. In the network, Talin-1 was found to promote HCC growth via suppressing the expression of apoptosis factors in the p53 network and increasing the expression of anti-apoptotic members of the BCL2 family. It also promotes HCC metastasis by increasing the expression of EMT mesenchymal markers and inhibiting the expression of epithelial molecules. This report comprehensively analyzed the mechanisms by which Talin-1 promotes HCC progression; our findings not only constitute a valuable resource for further investigation of Talin-1 in HCC progression but also provide novel therapeutic targets for HCC.

After Talin-1 silencing, the ion transport and membrane depolarization were inactivated, and the principle genes involved in these two processes, such as CAV3 and KCNE3, were downregulated. Bioelectricity, electrical activity generated by living organisms, is essential for life processes in higher animals [25]. Currently, electrical signals are widely used in the clinic for diagnosis, e.g. electrocardiography (ECG), electroencephalography (EGG) and electromyography (EMG). Plasma membrane depolarization, a typical bioelectricity conducted primarily by ion channels, can trigger cell proliferation [25]. Changes in the state of ion channels (including Na^+^, Ca^2+^, K^+^, and CL^-^ channels) could regulate ion homeostasis and further affect plasma membrane depolarization. Increased Na^+^ and Ca^2+^ influx through ion channels is essential for membrane depolarization; meanwhile, increased K+ influx and Cl- efflux attenuates membrane depolarization [25]. In addition, cell migration and invasion require a concerted action of ion channels and migration-associated transporters [25]. As a proliferation and metastasis factor, targeting bioelectricity therapeutically bears great clinical potential. Ion transport proteins are easily accessible and often overexpressed or activated in cancer, which makes them attractive candidate targets for bioelectricity therapeutically.

In this study, many genes regulating these ion channels were found to change dramatically after Talin-1 knockdown. For example, CAV3 was downregulated after Talin-1 silencing, which could significantly activate N-type Ca^2+^ channels and in turn promote Ca^2+^ influx [25]. Meanwhile, KCNE3 was upregulated after Talin-1 knockdown, which could significantly increase K^+^ efflux and Cl^-^ influx via interacting with KCNQ1. These studies suggest that Talin-1 may induce ion transformation and eventually promote membrane depolarization.

Overall, Talin-1 may promote HCC growth and metastasis via regulating cellular electrical activities and act as a potential target of bioelectricity therapeutically.

Unchecked growth and apoptosis avoidance are hallmarks of cancer cells, with the p53 network and BCL-2 family being their typical markers [25]. According to the above microarray data, after Talin-1 silencing, the expression of most cell growth-related genes which regulate the p53 network and BCL-2 family, including EDN1 and CYR61, were significantly decreased.

EDN1, a well-known cell cycle inducer in the p53 network, is overexpressed in human HCC [26]. In a zebrafish model, Lu Jw et al. demonstrated that EDN1 triggers hepatocarcinogenesis and promotes cell proliferation via AKT signaling [27]. Meanwhile, CYR61, a growth factor-inducible gene, is overexpressed in HCC and positively correlated with increased venous invasion and recurrence [28]. Also, CYR61 overexpression promotes cell proliferation by decreasing p21, p53, and Bax expression levels and increasing Bcl-xL, Mcl-1, Bcl-2 and NF-κB [25].

In conclusion, these findings demonstrate that Talin-1 promotes cell proliferation by affecting the expression of BCL-2 family and p53 network.

Talin-1 interacts with a large number of adhesion molecules (e.g. integrin and F-actin). Whether Talin-1 is involved in adhesion in other ways remains unclear. Here, we found that the expression of genes involved in cell adhesion present a downward trend after Talin-1 silencing, which suggests that Talin-1 can promote cell adhesion.

WISP1 encodes WNT1 inducible signaling pathway protein 1, which acts as an oncogene in HCC [29]. Multiple studies have reported that WISP1 can stimulate cell adhesion [30, 31]. In addition, both WISP1 and MUC1 were reported to promote cell adhesion via activating EMT process. Our findings suggest that Talin-1 could interact with multiple adhesion molecules, verifying and supplementing previous studies.

Currently, immunotherapeutic strategies have attracted increasing attention for the treatment of patients with relapsed or refractory cancer. Interestingly, the anti-SLAMF7 antibody-elotuzumab has been approved for use by Food and Drug Administration (FDA), and enhances natural killer cell activation and cancer cell killing through interleukin-2 and TNF-α pathways, significantly improving progression-free survival of multiple myeloma [32–34].

Talin-1 may promote HCC metastasis via promoting cell adhesion, and it is a potential target for immunotherapeutic strategies. Although the main biological behaviors and network of Talin-1 in cancer progression were analyzed in this study, other biological behaviors may be also important for HCC progression and deserve further attention.

Overall, Talin-1 acts as a proto-oncogene in HCC by activating ion transport, membrane depolarization, cell growth and cell adhesion, altering the expression of BCL-2 family and EMT-related genes, among others.

## MATERIALS AND METHODS

### Cell lines and culture

Established HCC (MHCC-97L) and normal liver epithelial (LO2) cell lines were obtained from Keygentec Inc. (Nanjing, China) and Basic Medical of AnHui Medical University (Hefei, China), respectively. They were cultured in DMEM (HyClone, Logan, USA) and RPMI-1640 (HyClone, Logan, USA), respectively, supplemented with 10% fetal bovine serum (FBS, Gibco, Paisley, UK).

### Quantitative polymerase chain reaction (PCR) and Western blot

Quantitative PCR (qRT-PCR) and Western blot were performed as previously described [35, 36]. Total RNA was extracted with Trizol reagent (Invitrogen). cDNA was obtained using RNA with PrimeScript RT-polymerase (Takara) and amplified using SYBR Green Mix (Takara Bio, Dalian, China). The cycling-threshold (CT) value for each gene was normalized to the level of β-actin, and the data was analyzed by the 2^-ΔCT^ method. The primers used in qRT-PCR are shown in Table 2. Anti-Talin-1 primary antibodies (ab104913, Abcam, Cambridge, MA, USA) were used for Western blot.

**Table 2:** Primers used in qRT-PCR

Gene	Accession NO.	Forward primer (5′—3′)	Reverse primer (5′—3′)
GAPDH	NM_002046.5	GGTCACCAGGGCTGCTTTTA	TTCCCGTTCTCAGCCTTGAC
CAV3	NM_001234	ACAAAGGCAACAGACCGTGA	GGTCTCCGACCTGGTTTGTC
AQP1	NM_198098	GCTTCAAATACCCGGTGGGG	TGTACATGAGGGCACGGAAG
PTGS2	NM_000963	ATAAGCGAGGGCCAGCTTTC	ACATCATCAGACCAGGCACC
CA9	NM_001216	CTGGTGACTCTCGGCTACAG	CTCATCTGCACAAGGAACGC
CLIC3	NM_004669	AGCTGTTTGTCAAGGCGAGT	TCTTGGCGTCGCTGTCATAG
KCNE3	NM_005472	CCAATGGAACGGAGACCTGG	GGTCACTACGCTTGTCCACT
EDN1	NM_001955	ACAAAGGCAACAGACCGTGA	GGTCTCCGACCTGGTTTGTC
CYR61	NM_001554	AATACCGGCCCAAGTACTGC	AGAAGGGAAACGCTGCTTCA
IGFBP5	NM_000599	GTCCAAGTTTGTCGGGGGAG	GGGAAGGTTTGCACTGCTTT
IGFBP3	NM_001013398	GCCCGCGCCAGGAAAT	TCGGAGGAGAAGTTCTGGGT
MUC1	NM_002456	AGTGCTTACAGTTGTTACGGGT	AGTAGTCGGTGCTGGGATCT
SLAMF7	NM_021181	GCCCCCATTCTGGAGAGAAC	ATAGCCTTGGTGTGTCTGGC
WISP1	NM_003882	GTAAGATGTGCGCTCAGCAG	CACGTGCAGTTGTACTTGCAG
FGB	NM_005141	AGCAGCTGCCACTCAAAAGA	GAGGAGGTCTGGGAAACAGC
VTN	NM_000638	CTCAAGGCCTGAGACCCTTC	CCTCACTGCCTTTTCGTCCA

### Establishment of a stable Talin-1 knockdown MHCC-97 L cell line

Lentiviral vectors were used for MHCC-97 L cell transduction, with puromycin employed for the selection of stable Talin-1 knockdown MHCC-97 L cells. The shRNA targeting the Talin-1 gene was 5′-GCTCGAGATGGCA AGCTTAAA-3′, and the nonspecific sequence (scramble shRNA) was 5′-TTCTCCGAACGTGTCACGTTTC-3′. Both transduction and selection were performed as previously described [25]. 0.5 × 10^5^ MHCC-97 L cells were cultured in 24-well plates using complete culture medium for 24 h. Afterward, these cells were transduced with lentivirus-mediated shRNAs for 72 h, under puromycin selection. Finally, these cells were cultured in complete culture medium with puromycin and stably transduced cells were obtained. Lentivirus titer was 1 × 10^8^ TU/ml, and a dilution of 1:9 was used. Puromycin was used at 3.0 μg/ml and adjusted to 1.5 μg/ml after one week. qRT-PCR and western blot were carried out to verify transduction efficiency.

### Proliferation and migration assays

Cell proliferation was assessed by cell counting. Briefly, 1.5 × 10^5^ cells were seeded into each well of a 12-well plate. After 1d, 2d, and 3d of incubation, respectively, the cells in each well were counted.

Transwell migration assay was performed as previously described [25]. A transwell chamber containing an 8-μm pore polycarbonate membrane filter (BD, USA) was used for the migration assay, with 1.5 × 10^5^ cells seeded in each well. Trans-membrane cells were dyed after 8 h.

### Gene expression analysis

Total RNA was extracted from MHCC-97L and sh-Talin-1 MHCC-97L cells and submitted to microarray analysis using Whole Human Genome Oligo Microarray (Agilent, G4112F). Image analysis was carried out with Agilent's Feature-Extraction V9.1.3 software. Fluorescence intensities were log_2_-transformed, and the SAS statistical software (http://sas.ebioservice.com/) was used for subsequent analyses. Differentially expressed genes were defined at a fold change cutoff of 2 and classified using the GO annotation (http://www.geneontology.org). GO project is used to help interpret the results of microarrays, using the Fisher's exact test to annotate and classify differentially expressed genes according to their function (cellular component, molecular function, and biological process) [37, 38]. Fisher's exact test is a method used to compute *P-value* based on hypergeometric distribution.

Hierarchical clustering (Complete Linkage Clustering) and heat maps were generated with the Multiple Experiment Viewer 4.9 (MEV 4.9) software. A scatter plot was generated using GraphPad Prism 5.

### Establishment of interaction network

The downstream target genes for all selected genes in microarray data were identified by searching published reports and two online bioinformatics databases, including STRING (http://string-db.org) and GeneMANIA (http://genemania.org/). Interaction network for the genes were created using the open source Bioinformatics software Cytoscape v3.4.0 (http://www.cytoscape.org/).

### Statistical analyses

Data are presented as mean ± standard error of arithmetic mean (SEM). Two-tailed unpaired Student's *t-test* was used for group comparison. *P <* 0.05 was considered statistically significant.
